# Comparative analysis of antibiotic resistance and biofilm formation in *Enterococcus* spp. across One Health domains

**DOI:** 10.1093/femsmc/xtaf005

**Published:** 2025-04-25

**Authors:** Vanessa Silva, Catarina Freitas, Jessica Ribeiro, Gilberto Igrejas, Patricia Poeta

**Affiliations:** LAQV-REQUIMTE, Department of Chemistry, NOVA School of Science and Technology, Universidade Nova de Lisboa, 1099-085, Caparica, Portugal; Department of Genetics and Biotechnology, University of Trás-os-Montes and Alto Douro (UTAD), 5000-801, Vila Real, Portugal; Functional Genomics and Proteomics Unit, University of Trás-os-Montes and Alto Douro (UTAD), 5000-801, Vila Real, Portugal; Microbiology and Antibiotic Resistance Team (MicroART), Department of Veterinary Sciences, University of Trás-os-Montes and Alto Douro (UTAD), 5000-801, Vila Real, Portugal; Department of Genetics and Biotechnology, University of Trás-os-Montes and Alto Douro (UTAD), 5000-801, Vila Real, Portugal; Functional Genomics and Proteomics Unit, University of Trás-os-Montes and Alto Douro (UTAD), 5000-801, Vila Real, Portugal; Microbiology and Antibiotic Resistance Team (MicroART), Department of Veterinary Sciences, University of Trás-os-Montes and Alto Douro (UTAD), 5000-801, Vila Real, Portugal; LAQV-REQUIMTE, Department of Chemistry, NOVA School of Science and Technology, Universidade Nova de Lisboa, 1099-085, Caparica, Portugal; Department of Genetics and Biotechnology, University of Trás-os-Montes and Alto Douro (UTAD), 5000-801, Vila Real, Portugal; Functional Genomics and Proteomics Unit, University of Trás-os-Montes and Alto Douro (UTAD), 5000-801, Vila Real, Portugal; Microbiology and Antibiotic Resistance Team (MicroART), Department of Veterinary Sciences, University of Trás-os-Montes and Alto Douro (UTAD), 5000-801, Vila Real, Portugal; LAQV-REQUIMTE, Department of Chemistry, NOVA School of Science and Technology, Universidade Nova de Lisboa, 1099-085, Caparica, Portugal; Department of Genetics and Biotechnology, University of Trás-os-Montes and Alto Douro (UTAD), 5000-801, Vila Real, Portugal; Functional Genomics and Proteomics Unit, University of Trás-os-Montes and Alto Douro (UTAD), 5000-801, Vila Real, Portugal; LAQV-REQUIMTE, Department of Chemistry, NOVA School of Science and Technology, Universidade Nova de Lisboa, 1099-085, Caparica, Portugal; Microbiology and Antibiotic Resistance Team (MicroART), Department of Veterinary Sciences, University of Trás-os-Montes and Alto Douro (UTAD), 5000-801, Vila Real, Portugal; Associate Laboratory for Animal and Veterinary Sciences (AL4AnimalS), Portugal; CECAV – Veterinary and Animal Research Centre, University of Trás-os-Montes and Alto Douro, 5000-801, Vila Real, Portugal

**Keywords:** *Enterococcus*, antimicrobial resistance, biofilm formation, One Health

## Abstract

The rise of antibiotic-resistant bacteria is a critical issue across various ecological interfaces, highlighting the need for a One Health approach. *Enterococcus* spp., known for their ability to acquire and disseminate resistance, serve as an excellent model due to their presence in diverse hosts and environments. This study investigates antimicrobial resistance, biofilm formation capacity, and the efficacy of antibiotics on biofilm biomass reduction in isolates from multiple sources. A total of 197 *Enterococcus* isolates were used. Antimicrobial resistance was determined using the Kirby-Bauer disc diffusion method, and minimum inhibitory concentrations were tested against vancomycin, tetracycline, and ampicillin. Biofilm formation capacity was assessed, and 10 biofilm-formers were subjected to minimum biofilm inhibitory concentration (MBIC) tests to evaluate biofilm biomass reduction. The results showed high resistance rates to erythromycin (84.5%), ciprofloxacin (59.4%), and tetracycline (44.4%), with moderate resistance to ampicillin (36.2%), chloramphenicol (28%), and vancomycin (24.7%). Biofilm formation was observed in 65% of the isolates, with *Enterococcus hirae* producing the most biofilm biomass. Vancomycin and ampicillin were more effective in reducing biofilm biomass than tetracycline. Ampicillin-resistant isolates produced more biofilm, suggesting a link between resistance and biofilm formation. This study highlights the complexity of antibiotic-resistant *Enterococcus* spp. and their biofilms, emphasizing the need for research on One Health.

## Teaser

This study reveals that antibiotic-resistant Enterococcus bacteria from diverse sources can form biofilms, making infections harder to treat and highlighting the urgent need for new strategies to combat these resilient pathogens.

## Introduction

*Enterococcus* species are ubiquitous in nature, inhabiting a wide range of environments including soils, sediments, freshwater, marine water, beach sand, and various plants. They are also integral members of the gastrointestinal flora of both livestock and humans, commonly found in the intestines of healthy individuals and animals (Zaheer et al. 2020). In the human gastrointestinal tract, the most frequent species are *Enterococcus faecalis* and, to a lesser extent, *Enterococcus faecium*, while in food animals, *E. faecium, Enterococcus cecorum, E. faecalis*, and *Enterococcus hirae* are predominant (Klein 2003, Guzman Prieto et al. 2016). *Enterococcus* spp. are also commonly isolated from water contaminated by sewage or fecal wastes, making them reliable bacteriological indicators of fecal contamination (Zaheer et al. 2020). Despite their role as commensals, *Enterococcus* spp. are significant opportunistic pathogens. They are capable of forming biofilms on medical devices, such as catheters and other implants. Additionally, they are also responsible for various types of infections, including urinary tract infections, bacteremia, endocarditis, and infections at surgical sites (García-Solache and Rice 2019). The treatment of enterococcal infections is complicated by their increasing resistance to antibiotics, including vancomycin (Tyson et al. 2018a). Enterococci are known for their intrinsic resistance to several antimicrobials, such as penicillin, ampicillin, and most cephalosporins, and their capacity to acquire additional resistance determinants quickly (Ahmed and Baptiste 2017). This resistance extends to last-resort antimicrobials like quinupristin-dalfopristin, linezolid, daptomycin, and tigecycline (Niebel et al. 2015, Dubin and Pamer 2016, Ahmed and Baptiste 2017).

The ability of enterococci to form biofilms significantly enhances their survival and persistence in both clinical and environmental settings. Biofilm-associated infections are notoriously difficult to eradicate and serve as reservoirs for antibiotic-resistance genes (Ch'ng et al. 2019). *Enterococcus faecalis*, in particular, exhibits biofilm production that can increase resistance to antimicrobial agents and the immune response by up to 1000 times compared to nonbiofilm producers (Oli et al. 2022). Biofilms represent complex microbial communities where specific ecological microniches support the survival and growth of different organisms (Bryers 2008). In contrast to other well-studied biofilm-forming bacteria like *Pseudomonas aeruginosa* and *Bacillus subtilis*, the mechanisms of enterococcal biofilm formation are less understood (Ch'ng et al. 2019). Biofilms contribute significantly to the virulence and resilience of enterococci, facilitating persistent infections and environmental contamination (Woźniak-Biel et al. 2019). In healthcare settings, enterococcal biofilms pose unique challenges due to their ability to colonize a variety of surfaces, including medical implants (e.g. catheters, prosthetic valves), hospital equipment, and even nonsterile environments such as patient bathrooms or contaminated linens. These biofilms act as reservoirs of multidrug-resistant bacteria, increasing the likelihood of hospital-acquired infections (Lebreton et al. 2014, Oli et al. 2022). Their resistance to disinfection protocols and antimicrobial treatments complicates infection control and eradication efforts in hospitals. Furthermore, their widespread presence in the environment and the food chain underscores their role as sentinel organisms in antimicrobial resistance surveillance systems, as they can transfer resistant genes to other bacteria through horizontal gene transfer, posing risks to public health via contaminated food products (Tyson et al. 2018b).

While numerous studies have documented the antimicrobial resistance of *Enterococcus* spp. across different hosts and environments, there is limited understanding of how biofilm formation correlates with antimicrobial resistance in a One Health context. This holistic approach, which considers the interconnectedness of human, animal, and environmental health, is essential for comprehensively addressing antimicrobial resistance (Aslam et al. 2021). Moreover, the potential impact of biofilm-associated resistance on treatment outcomes in clinical and environmental settings remains poorly understood, underscoring the need for integrated research on this topic. The persistence, adaptability, and resistance of *Enterococcus* spp. highlight the need for ongoing research and targeted strategies to manage and mitigate their impact on public health. This study aims to deepen our understanding of *Enterococcus* spp. by investigating the antimicrobial resistance, minimum inhibitory concentrations (MIC), biofilm formation capacity and the efficacy of antibiotics on the reduction of biofilm biomass of isolates from multiple sources, thereby reinforcing the importance of a One Health approach to combat these resilient pathogens.

## Material and methods

### Bacterial isolates

A total of 197 *Enterococcus* spp. isolates were selected for this study. *Enterococcus* spp. were isolated from children with infection (*n* = 10), Lusitano horses (*n* = 10), ostriches (*n* = 10), chickens (*n* = 10), trouts (*n* = 10), cows (*n* = 10), gilthead seabream (*n* = 10), thrushes (*n* = 10), pigs (*n* = 10), ruminant cattle (*n* = 10), donkeys (*n* = 10), seagulls (*n* = 10), sea urchins (*n* = 10), lynx (*n* = 10), bats (*n* = 10), wolves (*n* = 10), eagles (*n* = 10), migratory birds (*n* = 10), wastewaters (*n* = 10), wastewater effluents (*n* = 7), and healthy children (*n* = 10) (Table 1). All *Enterococcus* spp. isolates included in this study were commensal strains, obtained from nonpathogenic sources within their respective hosts and environments. The strains were isolated using Slanetz-Bartley agar and Kanamycin Aesculin Azide Agar Base. The species were confirmed by PCR using specific primers and conditions (Marinho et al. 2013).

**Table 1.** Origin and species identification of *Enterococcus* isolates.

Enterococci species	Number of isolates	Isolation source
*E. faecium*	10	Healthy horses
*E. hirae*	10	Healthy ostriches
*E. faecium*	10	Healthy trouts
*E. faecalis*	10	Healthy chickens
*E. hirae*	10	Healthy cows
*E. faecalis*	10	Healthy gilthead seabream
*E. faecium*	10	Healthy thrushes
*E. faecium*	10	Healthy pigs
*E. faecalis*	7	Healthy ruminant cattle
*E. hirae*	3	Healthy ruminant cattle
*E. faecium*	10	Healthy donkeys
*E. faecium*	10	Healthy seaguls
*E. faecium*	10	Healthy sea urchins
*E. faecium*	10	Healthy linx
*E. faecalis*	10	Healthy bats
*E. faecalis*	10	Healthy wolves
*E. faecium*	10	Healthy eagles
*E. faecalis*	10	Healthy migratory birds
*E. faecium*	10	Wastewaters
*E. faecalis*	7	Wastewater effluents
*E. faecium*	10	Healthy children

### Antimicrobial susceptibility testing

Antimicrobial susceptibility was accessed using the Kirby-Bauer disc diffusion method and according to the Clinical & Laboratory Standards Institute (CLSI) guidelines. The antibiotics used were the following: ciprofloxacin, chloramphenicol, gentamycin, teicoplanin, vancomycin, tetracyline, erythromycin, and ampicillin. *Enterococcus faecalis* strain ATCC 29212 was used as quality control.

### Minimum inhibitory concentration

The MIC of vancomycin, ampicillin, and tetracycline was determined using the Broth Microdilution method, in accordance with the CLSI guidelines. The isolates were cultured in Muller-Hinton Broth (MHB) and incubated at 37°C, 150 rpm for 24 h, after which they were diluted in MHB with a 0.5 McFarland standard. Serial four- to five-fold dilutions of the antibiotics were prepared in 96 well microdilution plates, with each well inoculated with a bacterial suspension to achieve a total volume of 200 µl. The plates were incubated at 37°C, 150 rpm for 24 h, and the results were read using spectrophotometry at 630 nm to determine the MIC values.

### Biofilm formation

#### Biofilm formation assay

Biofilm formation was assessed using a modified version of the microtiter plate assay (Oniciuc et al. 2016). Briefly, two colonies from fresh enterococci cultures were inoculated into tubes with 3 ml of Tryptic Soy Broth (TSB, Oxoid Ltd.) and incubated at 37°C for 16 ± 1 h with continuous shaking at 120 rpm (ES-80 Shaker-incubator, Grant Instruments, Cambridge, UK). The enterococcal suspension was then standardized to a concentration of 10^6^ cfu/ml, and 200 µl of this suspension was added to each well of a 96-well plate. *Enterococcus faecalis* strain ATCC 29212 was used as a positive control, while uninoculated TSB served as the negative control. The plates were incubated at 37°C for 24 h under static conditions. Each experiment included seven technical replicates and was repeated three times in different weeks.

#### Biofilm biomass quantification

Biofilm biomass was measured using the Crystal Violet (CV) staining method, with slight modifications to the protocol described by Peeters et al. (2008). After incubation, wells were washed twice with 200 µl of distilled water to eliminate nonadherent bacterial cells and allowed to dry at room temperature for 2 h. Biofilm cells were fixed by adding 100 µl of methanol (VWR International) and incubating for 15 min at room temperature. The methanol was then removed, and plates were dried in a laminar flow cabinet for 10 min. Following this, 100 µl of 1% (v/v) CV solution was added to each well to stain the biofilm cells for 10 min at room temperature. Excess dye was removed by rinsing the plates with distilled water, and the stained biofilm cells were solubilized with 33% (v/v) acetic acid. Absorbance was measured at 570 nm using a BioTek ELx808U microplate reader (BioTek, Winooski, VT). Results were normalized against the biofilm formation of the positive control strain, *E. faecalis* strain ATCC 29212.

### Effect of antibiotics on 24-h old biofilms

To evaluate the impact of conventional antibiotics on biofilm mass reduction, 10 isolates were selected based on their strong biofilm formation capacity: four *E. hirae* strains from ostriches, two *E. faecalis* strains from wastewater effluents, one *E. faecalis* strain from gilthead seabream, one *E. faecium* strain from trout, one strain from seagulls, and one from eagles. The antibiotics vancomycin, tetracycline, and ampicillin were selected for this assay. Biofilm formation was conducted as outlined in the section “Antimicrobial susceptibility testing.” After forming 24-hour-old biofilms, the medium was aspirated and replaced with 200 µl of TSB supplemented with either one of the selected antibiotics at final concentrations of MIC, 5× MIC, and 10× MIC. The plates were then incubated at 37°C for 24 h under static conditions. Positive controls consisted of TSB without antibiotics. Following antibiotic incubation, biofilm mass was quantified using the CV staining method as described in the section “Biofilm biomass quantification.” Each experiment included four technical replicates and was conducted independently on two occasions.

### Statistical analysis

Descriptive statistics are presented as mean (M) and standard deviation (SD) where appropriate. Skewness and kurtosis coefficients were calculated to assess univariate normality. To evaluate the association between resistance, multiresistance phenotypes, and resistance to specific antimicrobials with biofilm formation, a one-way analysis of variance (ANOVA) followed by Tukey's post-hoc test and independent samples *t*-test were conducted. All statistical analyses were performed using SPSS (IBM SPSS Statistics 26). Effects were considered statistically significant at *P* < .05.

## Results and discussion

### Antimicrobial resistance

**Figure 1.**
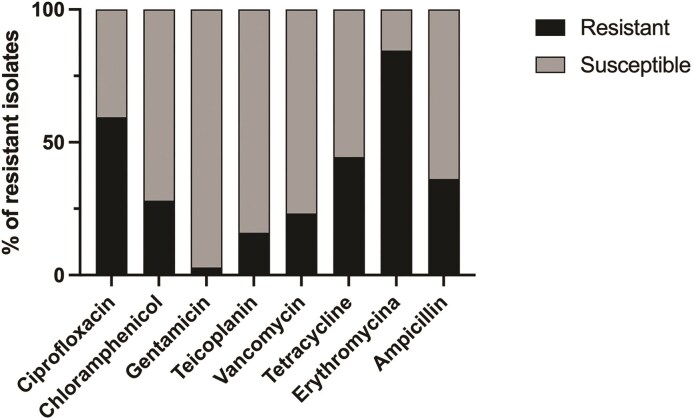
Percentage of antimicrobial resistance and susceptibility to each antibiotic of the 197 enterococcal isolates.

The emergence of antibiotic-resistant *Enterococcus* spp. poses a significant threat to public health, driven by their ability to thrive in diverse environments and hosts (Aslam et al. 2021, Cattoir 2022). Previous studies have highlighted the increasing prevalence of resistance to key antibiotics such as vancomycin, tetracycline, and ampicillin, as well as the formidable challenge presented by biofilm formation (Nath et al. 2020, Agyeman et al. 2022). Biofilms not only enhance bacterial survival and persistence but also complicate treatment efforts, making infections more difficult to eradicate (Cascioferro et al. 2021, Ma et al. 2022). In this study, we examined a collection of 197 *Enterococcus* isolates from various origins, comprising 110 *E. faecium*, 74 *E. faecalis*, and 23 *E. hirae*. All isolates were tested against eight antimicrobial agents belonging to seven different classes of antibiotics, and the overall results are shown in Fig. 1. Resistance to all tested antibiotics was observed among the isolates, with a high percentage showing resistance to erythromycin (84.5%), ciprofloxacin (59.4%), and tetracycline (44.4%). These findings are consistent with other reports on *Enterococcus* spp. isolated from humans, animals, and wastewaters (Novais et al. 2006, Kwon et al. 2012, Chakraborty et al. 2015, Haghi et al. 2019, Tian et al. 2019). Additionally, we detected moderate resistance rates to ampicillin (36.2%), chloramphenicol (28%), and vancomycin (24.7%), and lower resistance rates to teicoplanin (15.9%) and high-level gentamicin (2.9%). Nevertheless, resistance to gentamicin is typically higher in both human and animal isolates (Kwon et al. 2012, Diab et al. 2019, Cabral et al. 2020, Iancu et al. 2023). Overall, *E. faecalis* isolates exhibited a higher number of resistances compared to *E. faecium* or *E. hirae* isolates. Indeed, notably, 17 (15.5%) vancomycin-resistant isolates were *E. faecium* and 20 (27%) were *E. faecalis*. The World Health Organization has identified vancomycin-resistant enterococci (VRE) as a high-priority pathogen, highlighting the limited availability of effective treatment options. In this study, a moderate number of VRE were detected among our enterococci isolates, which is consistent with results from other European countries (European Centre for Disease Prevention and Control and World Health Organization 2023). Indeed, according to the ECDC report, in 2021, the presence of VRE in European countries varied from 1% to over 50%, with almost half of the countries reporting a frequency of 25% or higher (European Centre for Disease Prevention and Control and World Health Organization 2023).

**Figure 2.**
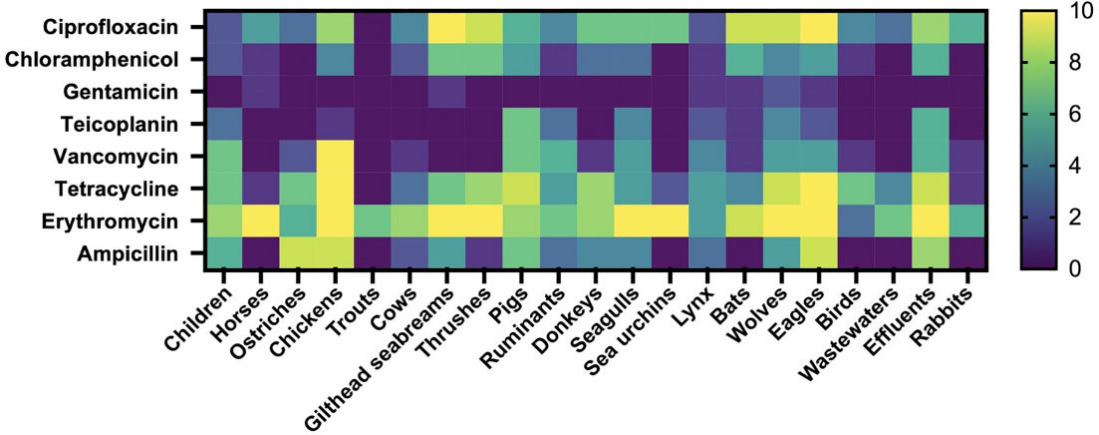
Heat-map of the number of isolates resistant to each antibiotic tested divided by the different sources.

The number of resistant isolates to each antimicrobial agent is shown in Fig. 2. Most strains isolated from livestock were resistant to tetracycline, which came as no surprise, since tetracyclines as well as penicillins are one of the antibiotics most frequently administered to these animals (Silva et al. 2020, Lianou and Fthenakis 2022). In our study, all isolates from chickens were VRE and also a high number of VRE were detected in pigs and effluents. In the 1990s, VRE were common in European farm animals. The ban on avoparcin in the late 1990s led to a significant decline in VRE prevalence. However, VRE clones are still found in poultry globally (Bortolaia et al. 2016, Leinweber et al. 2018). In this study, most wild animals carried enterococci with minimal antimicrobial resistance, likely because they live in natural environments and do not typically encounter antimicrobials (Radhouani et al. 2014, Grassotti et al. 2018). However, isolates from eagles and wolves showed resistance to a greater number of antibiotics, possibly due to their position at the top of the food chain and consumption of contaminated prey (Blanco et al. 2016, Baros Jorquera et al. 2021). Environmental contamination and the ingestion of animals that have been exposed to antibiotics are critical factors contributing to antimicrobial resistance in wildlife.

### Biofilm formation

**Figure 3.**
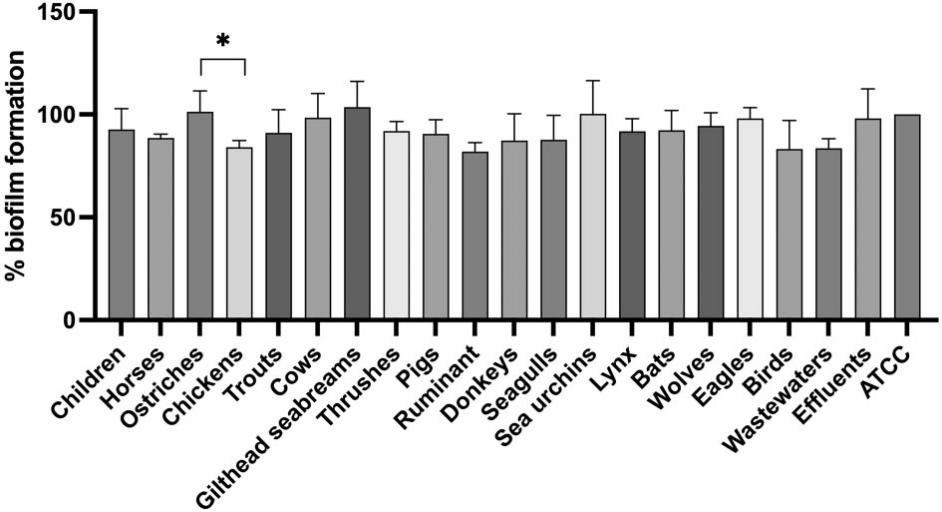
Percentage of biofilm formation of enterococci isolates. Each bar represents a different source of isolation.

Biofilms are complex communities of bacteria that adhere to surfaces and are encased in a protective extracellular matrix. Studying biofilm formation in *Enterococcus* spp. is crucial as it enhances bacterial survival in harsh environments and contributes significantly to their pathogenicity, including increased resistance to antibiotics and immune responses. This understanding can lead to better strategies for preventing and treating enterococcal infections. Biofilm formation was statistically compared among all isolates, taking into account their species and origin, to identify significant differences. All isolates were tested for their capacity to form biofilms. Out of the 197 isolates, 128 (65%) were classified as biofilm producers. The capacity for biofilm production in enterococcal isolates varies across studies. Some research indicates that all or nearly all enterococcal isolates can form biofilms, while other studies find that only a few isolates have this ability (Shridhar and Dhanashree 2019, Stępień-Pyśniak et al. 2019, Woźniak-Biel et al. 2019). In our study, among the 128 biofilm producers, 24 were classified as weak producers, 81 as moderate producers, and 23 as strong biofilm producers. Overall, enterococci isolates from ostriches, gilthead seabream, sea urchins, and effluents were the most biofilm producers (Fig. 3). Nevertheless, no significant differences in biofilm formation were observed between hosts, except for ostriches and chickens (*P* < .05). It is important to point out that all isolates from ostriches were identified as *E. hirae*. Other studies have shown that most *E. hirae* isolates are biofilm producers (Di Lodovico et al. 2017, Bino et al. 2018). Indeed, the biofilm formation ability of each *Enterococcus* species studied was also evaluated, revealing that *E. hirae* strains produce more biofilm biomass (101.3%) than *E. faecalis* (92.1%), and significantly more than *E. faecium* (91.7%) (*P* < .05). However, studies suggest that *E. faecalis* is generally considered a more robust biofilm producer compared to *E. faecium* and *E. hirae* (Mohamed and Huang 2007, Peng et al. 2017, Stępień-Pyśniak et al. 2019, Grudlewska-Buda et al. 2023). Despite the high number of antimicrobial resistances displayed by isolates from chickens, wolves, and eagles, these were not the most biofilm producers. Therefore, statistical tests were applied to verify whether antimicrobial resistance influences biofilm formation. Since only a small number of isolates showed resistance to chloramphenicol and gentamicin, these antibiotics were not considered for evaluating the influence of resistance on biofilm formation, as the low number of resistant isolates would result in an unbalanced analysis. Statistical analysis indicated a significant relationship between resistance to ampicillin and the biofilm-forming capacity of the isolates, with ampicillin-resistant isolates producing more biofilm (*P* = .0362) (Table 2). No correlation was found for tetracycline, vancomycin, erythromycin, ciprofloxacin, and teicoplanin, which is in accordance with the results obtained in other studies (Ramadhan and Hegedus 2005, Zheng et al. 2018, Al-Dahmoshi et al. 2019, Das et al. 2020, Ghazvinian et al. 2024).

**Table 2.** Mean (M), standard deviation (SD), and univariate effects of biofilm formation by resistant to each antibiotic.

Antibiotic	Resistant	Susceptible	*P*
	M±SD	M±SD	
Tetracycline	92.37 ± 9.83	94.43 ± 12.28	.3715
Vancomycin	96.05 ± 11.82	93.63 ± 10.83	.3037
Ampicillin	95.31 ± 10.56	91.28 ± 11.03	.0362
Erythromycin	94.39 ± 10.41	91.28 ± 11.03	.1149
Ciprofloxacin	94.36 ± 12.10	91.33 ± 10.00	.1260
Teicoplanin	92.32 ± 12.02	92.52+ 10.67	.9326

### Effect of antibiotics on 24-h old biofilms

**Figure 4.**
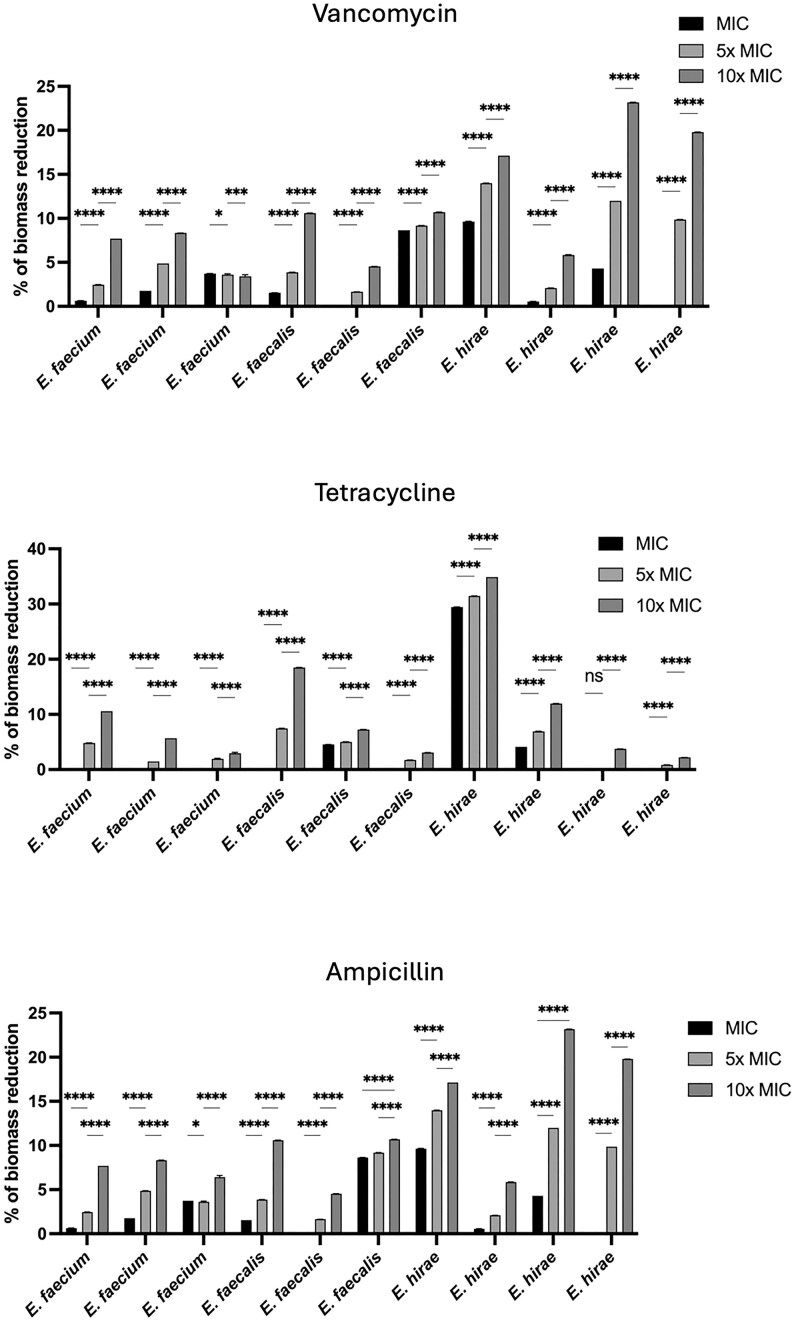
Percentage of biofilm biomass reduction caused by vancomycin, tetracycline, and ampicillin of 10 isolates at concentrations of MIC, 5x MIC, and 10x MIC. Statistical significance was determined using One-way ANOVA (*P < .05; ****P < .0001).

Biofilms enhance bacterial survival by providing a protective barrier against antimicrobial agents, leading to persistent and chronic infections (Gloag et al. 2020, Shree et al. 2023). Investigating the efficacy of antibiotics against *Enterococcus* biofilms is essential to develop more effective treatment strategies and combat the rising issue of antibiotic resistance in clinical settings (Mirghani et al. 2022, Sharma et al. 2023). In this study, 10 isolates (3 *E. faecium*, 3 *E. faecalis*, and 4 *E. hirae*) were selected to investigate the ability of antibiotics to reduce the biofilm biomass of mature biofilms using three antimicrobials at concentrations of MIC, 5× MIC, and 10× MIC. Out of the 197 *Enterococcus* isolates, 149 were susceptible to vancomycin, 105 to tetracycline, and 122 to ampicillin. The MICs for these susceptible isolates ranged from 0.5 to 4 µg/ml for vancomycin, 0.25–4 µg/ml for tetracycline, and 0.5–8 µg/ml for ampicillin. For the resistant isolates, the MICs ranged from 128 to >256 µg/ml for vancomycin, 16 to >256 µg/ml for tetracycline, and 16 to >256 µg/ml for ampicillin. The results are shown for the effect of vancomycin, tetracycline, and ampicillin on 24-hour-old biofilms are shown in Fig. 4. Overall, vancomycin achieved the highest biomass reduction at the MIC concentration. However, ampicillin was effective against all strains, unlike vancomycin, which did not reduce the biomass of two isolates. At the MIC concentration, tetracycline did not affect the biofilm biomass of seven isolates. At 5× MIC, all antibiotics had an mean of biofilm biomass reduction of around 6% and at 10× MIC the highest results were obtained with ampicillin with a mean of biomass reduction of 11.9%, followed by vancomycin (11.4%) and tetracycline (9.7%). Thus, antibiotics that target bacterial cell wall synthesis (vancomycin and ampicillin) had better efficacy on the reduction of biofilm biomass than tetracycline. This is contrary to what happens in staphylococci, as several studies have demonstrated that antibiotics that target protein or RNA syntheses have a higher efficiency than antibiotics that target cell wall synthesis, and this has been associated with the lower growth rate of cells within biofilms (Cerca et al. 2005, França et al. 2016, Carvalhais et al. 2017, Gaio and Cerca 2019). Studies have reported that tetracycline suppresses the localization of the autolysin Atl, which plays an important role in initial attachment of biofilm (Yamada et al. 2001, Ledala et al. 2006). Interestingly, in our study, although *E. hirae* strains produced more biofilm biomass than the other two species, all antibiotics had a greater effect on reducing the biomass of biofilms formed by *E. hirae*, particularly at 10× MIC. When considering the mean of biomass reduction to compare the efficacy of antibiotics on *E. faecium* and *E. faecalis*, there is a higher reduction in *E. faecalis* isolates with all antibiotics. Research by Dale et al. (2015) provided evidence that *E. faecalis* possesses genetic factors that facilitate antibiotic resistance within biofilms (Dale et al. 2015). Furthermore, the study also indicates that *E. faecalis* uses biofilm-specific mechanisms, rather than merely relying on extracellular matrix diffusion barriers, to protect against antibiotics (Dale et al. 2015).

## Conclusions

This study highlights the significant challenge posed by antibiotic-resistant *Enterococcus* spp. and their ability to form biofilms. The isolates exhibited high resistance to several antibiotics, including erythromycin, ciprofloxacin, and tetracycline, emphasizing the need for effective treatment strategies. Notably, ampicillin showed the highest efficacy in reducing biofilm biomass, particularly against *E. hirae*, which produced more biofilm than *E. faecalis* and *E. faecium*. A significant relationship between ampicillin resistance and increased biofilm production was observed, suggesting that resistance mechanisms may enhance biofilm formation. The differential effectiveness of antibiotics on biofilms formed by different *Enterococcus* species highlights the importance of targeted therapeutic approaches. Ongoing research and a One Health approach are essential to address the complex issue of antibiotic-resistant *Enterococcus* spp. and their biofilm-mediated infections. Future studies should focus on the genetic and molecular mechanisms underlying biofilm formation and resistance to develop more effective treatments.

## Supplementary Material

xtaf005_Supplemental_File
