# Supplementary material for: Comparative analysis of antibiotic resistance and biofilm formation in Enterococcus spp. across One Health domains
Source: FEMS Microbes. 2025 Apr 25;6:xtaf005. doi: 10.1093/femsmc/xtaf005 (PMC12077392; doi:10.1093/femsmc/xtaf005)
Supplement: xtaf005_Supplemental_File [file xtaf005_supplemental_file.docx]

This study reveals that antibiotic-resistant Enterococcus bacteria from diverse sources can form biofilms, making infections harder to treat and highlighting the need for new strategies to combat these resilient pathogens.
